# VEGF-A, -C, -D, VEGFR1, -2, -3, PDGF-BB and FGF-2 join forces to induce vascular and lymphatic angiogenesis during bone healing of hip implants

**DOI:** 10.1016/j.bonr.2025.101856

**Published:** 2025-07-02

**Authors:** Jean Cassuto, Agnetha Folestad, Jan Göthlin, Henrik Malchau, Johan Kärrholm

**Affiliations:** a Orthopedic Research Unit & Department of Orthopedic Surgery, Sahlgrenska University Hospital, Mölndal, Sweden; b Department of Radiology, Sahlgrenska University Hospital, Mölndal, Sweden; c Institution of Clinical Sciences, Göteborg University, Göteborg, Sweden; d Department of Orthopedic Surgery, Harvard Medical School, Boston, USA

**Keywords:** Bone regeneration, Vascular endothelial growth factor, Platelet derived growth factor, Placenta growth factor, Fibroblast growth factor-2, Hip arthroplasty

## Abstract

**Introduction:**

Angiogenic growth factors are a critical part of bone repair and regeneration in the aftermath of bone trauma. In the current study we monitored the spatiotemporal responses of angiogenic factors and receptors during the process of osseointegration of hip implants.

**Materials and methods:**

Twenty-four patients having undergone primary total hip arthroplasty (THA) due to one-sided osteoarthritis (OA) were monitored during a period of 18 years (Y) by repeated measurements of plasma biomarkers as well as clinical and radiographic variables, the latter two showing all implants of the study to be well anchored throughout the follow-up. Eighty-one healthy donors divided into three gender- and age-matched subgroups and twenty OA patients awaiting THA, served as controls. Plasma was analyzed for vascular endothelial growth factor (VEGF)-A, VEGF-C, VEGF-D, vascular endothelial growth factor receptor 1 (VEGFR1) or sFlt-1, VEGFR2 (KDR/sFlk-1), VEGFR3 (sFlt-4), platelet derived growth factor–BB (PDGF-BB), fibroblast growth factor-2 (FGF-2) and placental growth factor (PIGF). Analysis of biomarkers was done by means of a high-sensitivity and wide dynamic range electrochemiluminescence technique allowing for detection of low levels and minor changes in biomarker levels. Spatiotemporal changes of biomarkers in THA patients during the follow-up were presented in the context of the four phases of endochondral bone repair.

**Results:**

VEGF-A, VEGFR1, PDGF-BB and FGF-2 were significantly higher, whereas VEGF-C was significantly lower in presurgery OA patients versus healthy subjects but were all normalized shortly after implant surgery. VEGF-A, VEGF-C, VEGF-D, VEGFR2, VEGFR3, FGF-2 and PDGF-BB increased sharply 1–2 Y post-implant and reached a peak significantly above healthy control subjects at 5–7 Y after implant insertion before returning to control level 13-15Y post-surgery, except for VEGF-D that returned to control level at 7Y post-implant. VEGFR1 decreased to the level of healthy subjects at 6 W post-THA and remained there throughout the study. PIGF did not differ from healthy subjects at any point during the follow-up.

**Conclusion:**

Increased levels of VEGF-A, VEGFR1, PDGF-BB and FGF-2 and reduced VEGF-C in presurgery OA relative healthy subjects support a cartilage protective or disease-inducing role in osteoarthritis. The concerted increase by all proangiogenic factors of the study, except VEGFR1 and PIGF, at 5 Y post-implant lend strong support to this being the phase of bone repair when blood and lymph vessels invade the avascular calcified hypertrophic cartilage and trigger its remodeling into bone in hip arthroplasty patients.

## Highlights

• VEGF-A and VEGFR1 increased in OA and early after bone trauma.
• PDGF-BB and FGF-2 increased in OA and correlate to higher disease activity.
• VEGF-A and VEGFR2 induce vascular angiogenesis during endochondral bone repair.
• VEGF-C, —D and VEGFR2, -3 induce lymphatic angiogenesis during bone repair.
• PDGF-BB and FGF-2 are important during neoangiogenesis.

## 1 Introduction

The process of bone repair after bone trauma, accidental or surgical, requires the involvement of a significant number of biomolecules that form a complex web of actions and counteractions throughout the repair process aimed at the restoration of bone tissue (Einhorn and Gerstenfeld, 2015; Marsell and Einhorn, 2011). In cases when micromotion is present at the fracture site, as with hip implants, bone healing will proceed by endochondral repair that includes a cartilagenous phase (Einhorn and Gerstenfeld, 2015; Marsell and Einhorn, 2011). A key event during endochondral bone repair, without which the entire process will be stalled, is the ingrowth of newly formed blood vessels (angiogenesis) into the mineralized hypertrophic cartilage thereby initiating apoptosis of chondrocytes along with degradation of the cartilage matrix and its replacement with spongious bone (Hu and Olsen, 2016a). Angiogenesis during bone repair is induced by cells that release pro-angiogenic factors, such as VEGF, PDGF, FGF-2, PIGF and TGF-β, into the extracellular matrix (ECM) that will start the process of creating a new vascular network from preexisting vessels (Hu and Olsen, 2016a). VEGF-A, the most prominent angiogenic factor, is produced by hypertrophic chondrocytes, endothelial cells, macrophages and osteoblasts (Hu and Olsen, 2016a) and binds to two tyrosine kinase receptors, VEGFR1 and VEGFR2, on endothelial cells, osteoclasts and osteoblasts (Hu and Olsen, 2016a). In addition to being a potent angiogenic factor, VEGF-A has also been shown to promote chemotaxis of mesenchymal stem cells (MSC) that migrate into the hypertrophic cartilage and differentiate into osteoblasts (Hu and Olsen, 2016a). A primary role exerted by VEGF-A during angiogenesis is to induce vasodilation of preexisting capillaries or post-capillary venules thereby causing extravasation of plasma adhesion molecules that create a scaffold for the attachment of endothelial cell (EC) progenitors (Hu and Olsen, 2016a). This process requires the concomitant release of enzymes, like matrix metalloproteinases (MMPs), a disintegrin and metalloproteinase with thrombospondin motifs (ADAMTS) and serine proteases into the area of neovascularization (Roy et al., 2006) whereby they facilitate the disassembly of inter-endothelial cell adhesions, detachment of peri-endothelial smooth muscle cells and degradation of the basement membrane which allows EC to migrate into the surrounding ECM, proliferate and subsequently differentiate into lumenized endothelial sprouts (Hu and Olsen, 2016a). The endothelial cells forming the new vascular buds will then release growth factors, such as PDGF-BB, that attract so-called *pericytes*, i.e. smooth muscle cells and fibroblasts, that surround and stabilize the newly formed EC tubes and enable them to transport blood (Caplan and Correa, 2011). The role of the newly formed blood vessels is to function as a primary conduit of nutrients, immune cells, osteoclasts and osteoblasts that will release their contents of proinflammatory cytokines, enzymes and growth factors into the cartilage thereby enabling osteoblasts to fill the empty lacunae created by apoptotic chondrocytes and transform the cartilage into bone (Einhorn and Gerstenfeld, 2015; Marsell and Einhorn, 2011). The lymphatic system is functionally and structurally interconnected with the vascular system although the presence of lymph vessels in bone tissue has long been a matter of discussion (Huang et al., 2024). However, recent studies have confirmed the presence of a lymphatic network within the periosteum and long bones and elucidated their role in the maintenance and repair of the skeletal system (Huang et al., 2024). This has triggered a significant increase in research evaluating the role of two major growth factors that regulate the formation of lymphatic vessels, VEGF-C and VEGF-D, in bone homeostasis and repair (Huang et al., 2024).

The current study is based on the same population of hip arthroplasty patients and healthy subjects as three previous studies that had monitored the role of biomolecules during two decades following hip implant surgery and evaluated their role in the process of endochondral bone repair leading to a successful osseointegration of the implant (Cassuto et al., 2018; Cassuto et al., 2020; Cassuto et al., 2023). The aim of the present study was to investigate the role of angiogenic factors during bone healing of hip implants and discuss their role in bone repair (Einhorn and Gerstenfeld, 2015; Marsell and Einhorn, 2011).

## 2 Materials and methods

### 2.1 Study design and study population

The current study, with level of evidence II, was approved by the institutional review board of VästraGötalandsRegionen at Sahlgrenska University Hospital. Informed consent was obtained from every patient prior to inclusion. The study complies with the STROBE-statement for observational studies (von Elm et al., 2014). (I) Sixty consecutive patients scheduled for primary total hip arthroplasty (THA) due to one-sided osteoarthritis (OA) were enrolled into the study to evaluate different femoral stem designs by use of clinical variables, radiography, radiostereometry (RSA) and dual-energy x-ray absorptiometry (DEXA) (Karrholm et al., 2002; Thien et al., 2012). In addition, venous blood was sampled on each visit. The THA group received either of two uncemented stems i.e., Epoch®, a low-modulus stem with porous coating and reduced stiffness (*n* = 10) or Anatomic®, a titanium alloy stem with porous coating (*n* = 14), both from Zimmer-Biomet, Warsaw, Indiana, USA. Both stem types were supplied with an additional layer of hydroxyapatite and tricalciumphosphate (HA/TCP) on the porous coating. The Anatomic stems were also coated with pure hydroxyapatite distal to the porous coating. All patients received a cementless porous press-fit cup with HA/TCP coating (Trilogy®, Zimmer-Biomet) on the acetabular side that was fixed with or without additional screws. All cups were supplied with polyethylene liners, gamma-sterilized with 0.025 Gy in inert nitrogen gas. Characteristics, such as wear rates, stem migration patterns, loss of BMD and clinical outcome, were reported for the implants in a 7-year follow-up (Thien et al., 2012). Basic demographic data, comorbidities and medications were registered. Patients with a pre-existing hip implant prior to the index implant (*n* = 14) were excluded. Patients who developed clinical (see HHS and pain scores) and/or radiological signs of OA in the opposite hip joint during the follow-up (*n* = 12), were excluded. Eight patients with lysis or implant wear prompting revision surgery and two patients with clear-cut lysis, but no revision, were excluded. Patients diagnosed with prosthesis loosening during the current follow-up were not included in the current investigation as they proved to be too few to allow for a proper statistical evaluation. (I) The current study included twenty-four patients (mean age 58Y, range 40–69; 16 males and 8 females) that had received a one-sided total hip implant that was stable, judged by clinical and radiographic variables, throughout the follow-up of 18 Y without signs of OA in the contralateral hip. (II) Twenty OA patients awaiting THA (mean age 58Y, range 34–86; fourteen males and 6 females), were included as a control of the stability and validity of biomarkers in pooled plasma. Each biomarker was assessed by comparing levels in plasma stored for 18Y (presurgery sample, PR) with fresh plasma from OA patients awaiting THA. (III) Eighty-one healthy subjects were divided into three gender- and age-matched subgroups (mean age 58Y, 67Y and 79Y) that served as controls to THA patients over the course of the follow-up (Table 1) with age means of healthy individuals being chosen to represent an entry, a midpoint, and an exit point. Healthy subjects and OA patients awaiting THA were recruited between 10Y and 13Y after ending the inclusion of THA patients. Plasma from healthy subjects was analyzed within 5 months of sampling. We did not investigate socioeconomic status. Exclusion criteria in both the THA group, the OA group awaiting THA and the healthy groups were selected to minimize the risk of interference with the validity of biomarker results. We excluded subjects in all groups suffering from any kind of malignancy, immune disorder (e.g., HIV), immune-related joint diseases (e.g., rheumatoid arthritis) and diseases affecting the bone (e.g., Paget's disease), kidneys or liver. Individuals on steroids, immunotherapy, chemotherapy, or bone-regulating drugs (e.g., bisphosphonates, denosumab, calcitonin, PTH) were excluded. In addition, healthy subjects were also excluded if they had a history of bone trauma within a year of inclusion or diseases affecting the joints (e.g., osteoarthritis). Healthy subject, OA patients awaiting THA and patients that had undergone THA were not registered as smokers upon inclusion, although we cannot exclude that smokers in the latter group that were required to terminate smoking well ahead of surgery may have resumed their presurgery smoking habits during the follow-up.

**Table 1:** Demographic and routine laboratory data in the three groups of healthy subjects and in THA patients at inclusion. Comparisons between the three groups of healthy were made by one-way repeated measures ANOVA whereas comparison between healthy individuals (mean age 58Y) and the THA group at inclusion (mean age 58Y) were done by Wilcoxon rank sum test. Data were partly presented in a previous publication (Cassuto et al., 2020).

Subjects	Healthy (n = 27)	Healthy (n = 27)	Healthy (n = 27)	THA at inclusion (n = 24)	p-value comparison between healthy groups	p-value comparison of THA at inclusion vs. healthy age 58Y
Age (Y) mean ± SEM (range)	58 ± 1.7 (40–68)	67 ± 0.6 (61–72)	79 ± 1 (71–88)	58 ± 1.4 (40–69)	–	–
Gender (F/M)	11/16	8/19	7/20	8/16	–	–
Height (cm)	166 ± 7.6	177 ± 1.5	174 ± 1.5	171 ± 5	0.311	0.068
Weight (kg)	77 ± 4	87 ± 2	77 ± 3	83 ± 4	0.057	0.716
BMI (kg/m^2^)	25 ± 1	27 ± 1	26 ± 0.6	26 ± 1.1	0.587	0.820
Hb	133 ± 7	144 ± 2	145 ± 3	140 ± 4	0.800	0,867
WBC (x 10^9^/L)	6.2 ± 0.4	6.3 ± 0.5	6.5 ± 0.3	6.4 ± 0.3	0.364	0.506
Platelet count (x 10^9^/L)	216 ± 17	205 ± 10	216 ± 10	209 ± 12	0.344	0.589
S-creatinine (μmol/L)	85 ± 12	90 ± 2	91 ± 5	83 ± 8	0.285	0.505

### 2.2 Radiographs

Anteroposterior pelvic radiographs plus anteroposterior and axial radiographs of the THA hip and the contralateral hip were done in the THA group and the OA group awaiting THA. Radiographs in THA patients were taken shortly before surgery (PR), on day one post-surgery (PO = postoperative day1 or 1D = 1 day after surgery), 6 weeks (6 W), 3 months (3 M), 6 M, one year (1Y), 2Y, 5Y, 7Y, 10Y, 13Y, 15Y and 18Y after the index operation. Radiographs of the hip included the acetabular and femoral portions of the joint with examination for joint space narrowing, subchondral lucency, marginal osteophytes, and subchondral sclerosis. In cases with radiographic signs of OA in the contralateral hip joint, the patient was excluded due to potential interference with biomarkers of the joint representing the index operation.

### 2.3 Harris hip score (HHS) and pain scores

Harris Hip Score (Harris, 1969) was measured before surgery (PR), 1Y, 2Y, 3Y 5Y, 7Y, 10Y, 13Y, 15Y and 18Y after surgery and supplemented with a more detailed questionnaire of hip pain used to monitor subjective variables of hip function (Table 2). The HHS is a 13-item patient/clinician report of pain (44-points); function (47-points); deformity (4-points); pre- and postoperative patient reported outcome measures (PROM) (5-points). A visual analogue scale is used and then scaled to a 100-point sum (maximum perfect score = 100). Results can be interpreted with the following: <70 = poor result; 70–80 = fair, 80–90 = good, 90–100 = excellent. Pain scores were graded as follows: 44 (no pain or negligible pain), 40 (mild pain but no functional disability), 30 (no ADL dysfunction but mild pain in connection with physical activity prompting occasional use of analgesics), 20 (ADL limited by constant moderate pain necessitating the use of analgesics on regular basis), 10 (severe pain with pronounced limitation of ADL and regular use of analgesics), 0 (severe and disabling pain at rest with continuous use of analgesics).

**Table 2:** Harris hip score (HHS) and pain scores in THA patients. PR = preoperative, 1 Year (Y) to 18Y after surgery. ***p < 0.001 PR versus 1Y post-THA (Wilcoxon rank sum test). Data have been previously published (Cassuto et al., 2018; Cassuto et al., 2020; Cassuto et al., 2023).

HHS	PR	1Y	2Y	3Y	5Y	7Y	10Y	13Y	15Y	18Y
Median Range	54 25–75	94*** 65–100	97 55–100	100 70–100	96 72–100	98 51–100	100 50–100	96 84–100	100 86–100	95 89–100

Pain										
Median Range	20 0–30	44*** 20–44	44 10–44	44 20–44	44 30–44	44 30–44	44 30–44	44 30–44	44 40–44	44 44–44

### 2.4 Blood sampling and analysis of plasma biomarkers

Venous blood was drawn into EDTA tubes from THA patients on the following occasions: one day before surgery (PR), one day after surgery (PO or1D), 6 W, 3 M, 6 M, 1Y, 2Y, 5Y, 7Y, 10Y, 13Y, 15Y and 18Y post-surgery. In the three groups of healthy controls and the group of OA patients awaiting THA, venous blood for cytokine analysis was sampled on a single occasion. Blood was sampled during normal working hours. Blood samples for cytokine analysis were centrifuged at 4 °C and immediately stored at -85 °C until analysis. Plasma biomarkers were analyzed on a high-sensitivity and wide-dynamic range platform from MesoScaleDiagnostics (Sector Imager 2400®; Rockville, Maryland, USA; for details see www.mesoscale.com). Precoated 96-well plates from MSD were used for plasma analysis of VEGF-A, PIGF, FGF-2 and VEGFR1 (Human Growth Factor panel I, K151IUC). Matched pairs of antibodies, i.e., capture antibody (CA) and biotinylated detection antibody (DA) labeled with streptavidin SULFO-TAG®, were used for analysis of plasma biomarkers as specified below. Standard curves were created using human recombinant proteins (hRP). Plasma was mounted on uncoated standard plates from MSD (L15XA). Antibodies were used within 5 months of arrival to our lab. VEGF-C, CA: anti-human monoclonal mouse IgG2_B_ antibody (Biotechne R&D Systems, cat.no.MAB752), DA: biotinylated antigen affinity-purified polyclonal goat IgG antibody (Biotechne R&D Systems, cat.no.BAF752), hRP (Peprotech, cat.no. 100-20C). VEGF-D, CA: affinity-purified polyclonal goat IgG antibody (Biotechne R&D Systems, cat.no.AF286), DA: biotinylated antigen affinity-purified polyclonal goat IgG antibody (Biotechne R&D Systems, cat.no.BAF286), hRP (Peprotech, cat.no. 100-20D). VEGFR2, CA: antigen affinity-purified polyclonal goat IgG antibody (Biotechne R&D Systems, cat.no.AF357), DA: biotinylated antigen affinity-purified polyclonal goat IgG antibody (Biotechne R&D Systems, cat.no.BAF357), hRP (MSD, Growth factor 2-plex assay: KDR & cKit). VEGFR3, CA: monoclonal mouse IgG_1_ (Biotechne R&D Systems, cat.no.MAB349), DA: biotinylated monoclonal mouse IgG_1_ (Biotechne R&D Systems, cat.no.BAM3492), hRP (Biotechne R&D Systems, cat.no.349-F4). PDGF-BB, CA: antigen affinity-purified rabbit antibody (Peprotech 900-K04), DA: biotinylated antigen affinity-purified rabbit antibody (Peprotech cat.no.900-K04), hRP (Peprotech cat.no.900-K04). Antibodies were optimized by checkerboard titrations and subsequent control of standard curves. Inter-assay variations were < 5 %.

#### 2.4.1 Stability of stored plasma

An important issue when storing blood samples over extended periods of time is the degree of degradation that could have significant impact on the interpretation of data. To ascertain the validity of stored plasma, we compared plasma levels of biomarkers in THA patients taken before surgery and stored for 18 years with levels in fresh plasma (not older than 5 months) from OA patients awaiting THA. Inter-group variability was minimized by mounting plasma from both groups on the same analytical plates. To safeguard the stability of biomarkers over the course of time, EDTA tubes containing frozen plasma (-85 °C) were thawed on ice in limited numbers before being aliquoted into cryotubes (120 μl/tube) and stored at -85 °C for short periods of time before use. Each cryotube was only thawed and used on a single occasion for analysis on four different plates (25 μl/well, see www.mesoscale.com) with excessive plasma being discarded. Evaluations at our lab showed no distinguishable changes in biomarker levels after 3 freeze/thaw cycles.

### 2.5 Statistical analysis

**Fig. 1:**
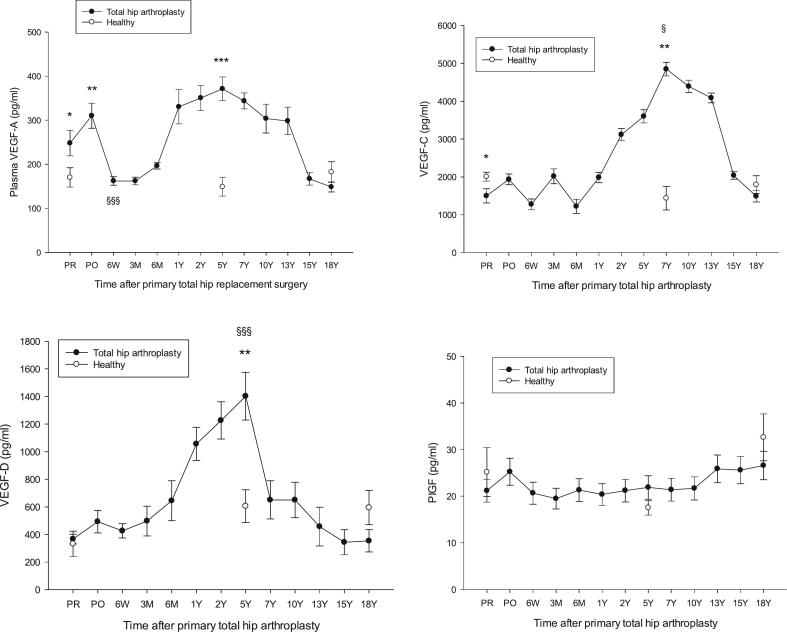
VEGF-A, VEGF-C, VEGF-D, and PIGF in patients that had undergone a primary THA versus healthy controls, VEGF-A, *p = 0.011 THA PR vs healthy 58Y, **p = 0.009 THA PO vs healthy 58Y, ***p < 0.001 THA 5Y vs healthy 67Y, ^§§§^*p* = 0.02 THA PO vs THA 6 W. VEGF-C, *p = 0.034 THA PR vs healthy 58Y, **p = 0.002 THA 5Y vs healthy 67Y, ^§^*p* = 0.019 THA PR vs THA 5Y. VEGF-D, **p = 0.009 THA 5Y vs healthy 67Y, ^§§§^p < 0.001 THA PR vs THA 5Y. PIGF, differences between healthy individuals and THA patients were not significant at any point of the follow-up. Other differences between THA and healthy individuals were not significant.

**Fig. 2:**
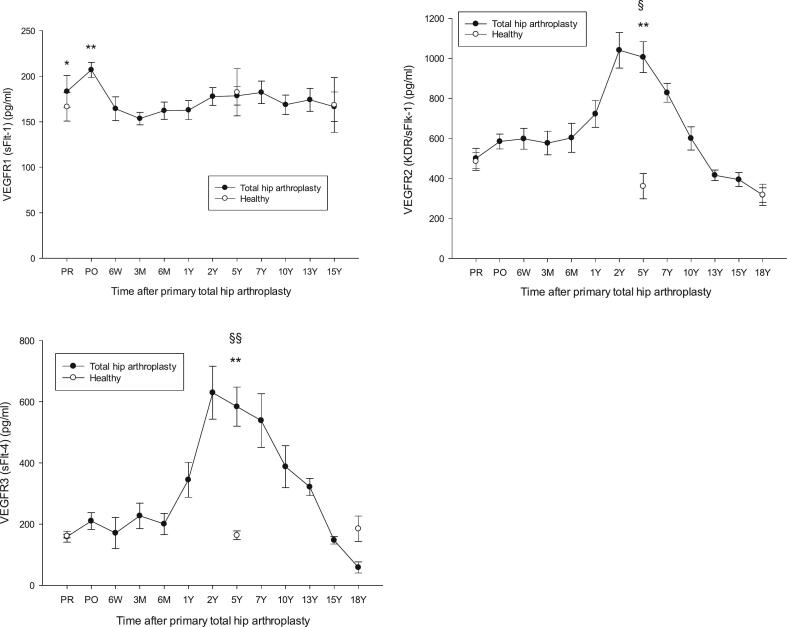
VEGFR1, VEGFR2 and VEGFR3 in patients having undergone primary total hip arthroplasty (THA) vs healthy controls, VEGFR1, *p < 0.05 THA PR vs healthy 58Y, **p = 0.004 THA PO vs healthy 58Y. VEGFR2, ^⁎⁎^*p* = 0.001 THA 5Y vs healthy 67Y, ^§^*p* = 0.014 THA 5Y vs THA PR. VEGFR3, **p = 0.004 THA 5Y vs healthy 67Y, ^§§^p = 0.019 THA PR vs THA 5Y. Other differences were not significant. Mean ± SEM.

**Fig. 3:**
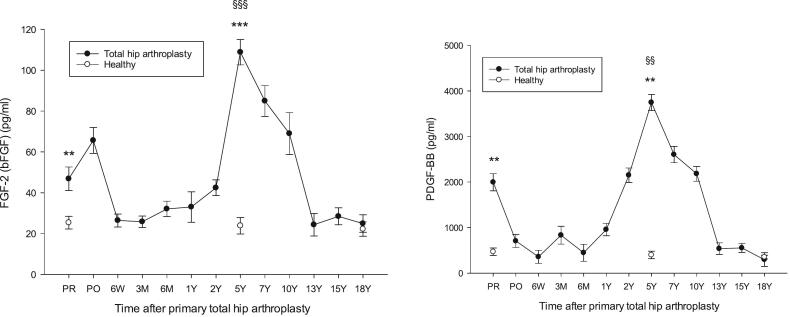
FGF-2 and PDGF-BB in patients having received a primary THA versus healthy controls. FGF-2, ^⁎⁎^*p* = 0.009 THA PR vs healthy 58Y, ***p < 0.001 THA 5Y vs healthy 67Y, ^§§§^ p < 0.001 THA 5Y vs THA PR. PDGF-BB, **p = 0.004 THA PR vs healthy 58Y, p = 0.009 THA 5Y vs healthy 67Y. Other differences were not significant. Mean ± SEM.

One-way repeated measures ANOVA with post hoc Holm-Śidak test was used to analyze differences in demographic and laboratory data (Table 1). Comparison of differences in individual biomarker levels between individuals in the three age groups of healthy was done by means of one-way repeated measures ANOVA (Table 3). One-way analysis of variance (ANOVA) with post hoc Holm-Śidak test was used to compare biomarkers in the THA group, representing a continuous dependent variable (time), versus the independent categorical groups of healthy subjects (Fig. 1, Fig. 2, Fig. 3). Normality was assessed by Shapiro-Wilk and Kolmogorov-Smirnov tests. Equal variance was assessed by the Brown-Forsythe test. Log transformation was used to normalize data when necessary. Age-matched comparisons between the three age groups of healthy (58Y, 67Y and 79Y) and the corresponding age intervals in the THA group were performed as follows: 1) THA PR vs. healthy mean age 58Y (all biomarkers), 2) THA PO, 3 M, 6 M, 1Y and 2Y vs. healthy mean age 58Y (all biomarkers), 3) THA representing high activity between 5Y and 13Y vs. healthy mean age 67Y (all biomarkers), 4) THA 15Y or 18Y vs. healthy mean age 79Y (all biomarkers). Comparison of biomarker levels within the THA group were done between PR (mean age 58Y) and 5Y (mean age 67Y) and between PR (mean age 58Y) and 18Y (mean age 79Y) by Wilcoxon rank sum test. Evaluation of stability of biomarker levels in stored plasma was done by comparison of plasma sampled before surgery (stored 18Y) in the THA group (mean age 58Y) with fresh plasma from age-matched individuals in the OA group awaiting THA (mean age 58Y) by means of Wilcoxon rank sum test (Table 4). Data are presented as the mean ± SEM.

**Table 3:** Angiogenic factors in the three groups of healthy controls. Mean ± SEM.

Age (Y) (range)	Healthy 58 ± 1.7 (40–68)	Healthy 67 ± 0.6 (61–72)	Healthy 79 ± 1 (71–88)	*p*-values
VEGF-A (pg/ml)	25 ± 3	24 ± 4†	22 ± 3*§	†*§ = 0.834
VEGF-C (pg/ml)	2006 ± 116	1436 ± 310 †	1794 ± 232 *§	†*§ = 0.222
VEGF-D (pg/ml)	331 ± 91	605 ± 118 †	594 ± 123 *§	†*§ = 0.378
VEGFR1 (pg/ml)	166 ± 15	182 ± 25 †	168 ± 30 *§	†*§ = 0.677
VEGFR2 (pg/ml)	485 ± 44	361 ± 63 †	317 ± 53 *§	† = 0.007 § = 0.003
VEGFR3 (pg/ml)	161 ± 6	163 ± 14 †	185 ± 41 *§	†*§ = 0.199
PDGF-BB (pg/ml)	469 ± 83	399 ± 81 †	351 ± 41 *§	†*§ = 0.292
FGF-2 (pg/ml)	25 ± 3	23 ± 4 †	22 ± 4 *§	†*§ = 0.866
PIGF (pg/ml)	25 ± 5	17 ± 1 †	32 ± 5 *§	* = 0.039

One-way repeated measures ANOVA was used for comparison of: † Healthy 67 Y versus healthy 58 Y, ^⁎^ Healthy 79 Y versus healthy 67 Y, ^§^ Healthy 79 Y versus healthy 58 Y. †*§ Inter-group differences not significant.

**Table 4:** Angiogenic factors in preoperative THA patients vs. osteoarthritis (OA) patients awaiting THA. Mean ± SEM.

Age (Y) mean ± SEM (range)	Preoperative THA 58 ± 1.7 (40–68)	Presurgery OA awaiting THA 58 ± 1 (34–86)	*p*-value OA vs THA
VEGF-A (pg/ml)	46 ± 5	49 ± 11	0.273
VEGF-C (pg/ml)	1396 ± 187	1541 ± 159	0.106
VEGF-D (pg/ml)	366 ± 35	380 ± 131	0.401
VEGFR1 (pg/ml)	186 ± 16	201 ± 23	0.167
VEGFR2 (pg/ml)	536 ± 51	460 ± 27	0.586
VEGFR3 (pg/ml)	159 ± 17	188 ± 23	0.441
PDGF-BB (pg/ml)	1995 ± 187	2017 ± 203	0.383
FGF-2 (pg/ml)	46 ± 5	49 ± 11	0.273
PIGF (pg/ml)	21 ± 1	23 ± 4	0.542

## 3 Results

Comparison of demographic and routine laboratory data between the three groups of healthy controls and between healthy controls (mean age 58Y) and THA-patients at inclusion (mean age 58Y) were not significant (Table 1). HHS and pain scores presented in Table 2 have been presented in previous studies from the current population of THA patients (Cassuto et al., 2018; Cassuto et al., 2020; Cassuto et al., 2023). Age-related changes in biomarker levels between the three groups of healthy are presented in Table 3. Levels of individual biomarkers in plasma of THA patients taken before surgery (PR) and stored for 18Y showed no significant differences versus the corresponding biomarker levels in fresh plasma from OA patients awaiting THA (Table 4).

**Fig. 4:**
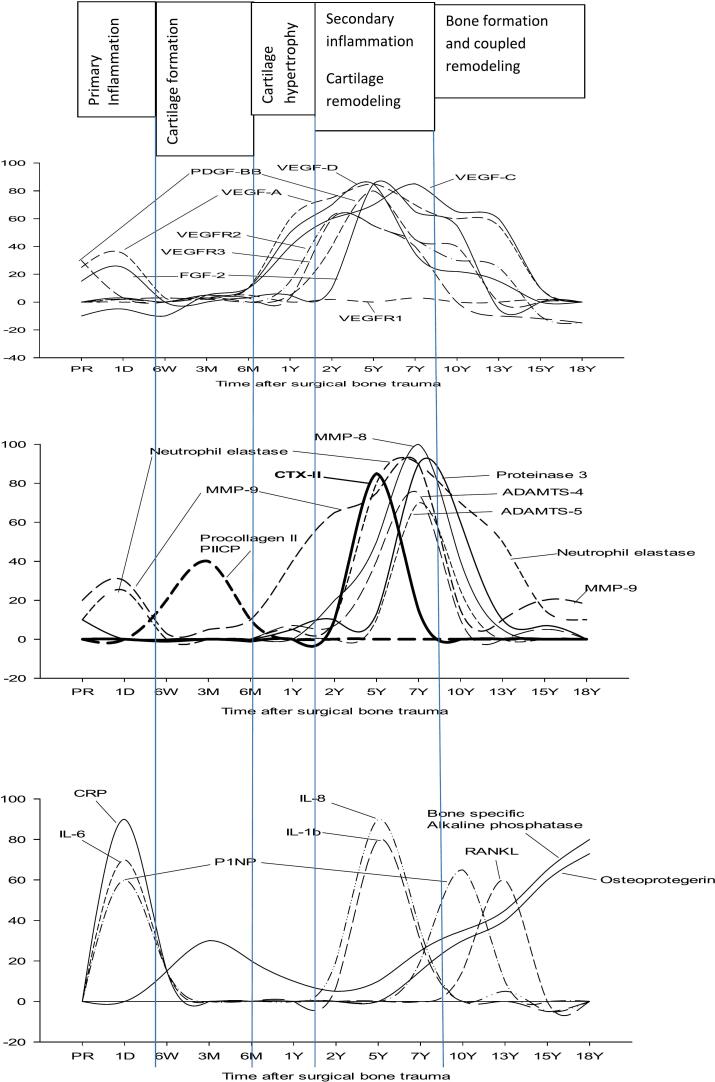
Schematic presentation of the major phases of endochondral bone repair and the spatiotemporal presentation of important pathways that regulate osseointegration of hip implants. All the biomarkers in the figure originate from the same population of patients that had undergone a primary total hip arthroplasty (THA) and were monitored by repeated sampling and analysis of biomarkers over the course of two decades. For the sake of clarity, biomarker data were presented in separate previous publications that focused on specific functional areas of biomarker activity during bone repair. Increased plasma levels of biomarkers that occurred during the course of bone repair and were significantly above the levels of biomarkers in age- and gender-matched healthy subjects are presented as upward deflections from zero level (=0) which represents the level of healthy controls. The height of individual biomarker graphs do not reflect the actual increase in plasma level of biomarkers in THA patients relative to controls. The **upper panel** is a summary of the angiogenic biomarkers of the current study that showed significant increase relative healthy control subjects. The **center panel** represents the spatiotemporal changes of enzymes that are involved in the degradation and remodeling of the calcified hypertrophic cartilage callus as shown by the sharp increase by the marker of type II collagen degradation biproducts peaking at 5Y post-implant (**CTX-II**, thick line) and coinciding with the peaks of enzymes involved in cartilage remodeling, i.e. MMPs, ADAMTS and serine proteases. Cartilage remodeling was preceded by peak level of cartilage formation markers (**procollagen II** and **PIICP**) at 3 months post-implant. Original data from the center panel have been presented in a previous publication (Cassuto et al., 2020). The **lower panel** shows the spatiotemporal changes of proinflammatory cytokines and biomarkers involved in bone repair and remodeling (Cassuto et al., 2018). They show the spatiotemporal changes of **P1NP** and **ALP** representing bone formation markers and **RANKL** representing osteoclastogenesis. **Acronyms**: Upper panel: see text. Center panel: PIICP (procollagen II of terminal propeptide), CTX-II (C-terminal telopeptide fragments of type II collagen), MMP (matrix metalloproteinase), ADAMTS (a disintegrin and metalloproteinase with thrombospondin motifs). Lower panel: CRP (C-reactive protein), IL (interleukin), P1NP (N-terminal propeptide of collagen type I), RANKL (receptor activator of nuclear factor kappa B-ligand). **Summary**: The peaks of VEGFs, VEGFRs, PDGF and FGF-2 at 5Y post-implant align with the peaks of proinflammatory cytokines (IL-8, IL-1b; lower panel) and enzymes involved in the remodeling of the cartilage into bone (MMPs, ADAMTS, serine proteases; center panel) thus supporting the concerted actions of angiogenic, enzymatic and proinflammatory mediators during the phase of cartilage remodeling and its transformation into bone.

Fig. 1, Fig. 2, Fig. 3 show levels of study biomarkers in THA and healthy controls throughout the follow-up. Fig. 4 summarizes the spatiotemporal changes of biomarkers of the current study and previous studies (Cassuto et al., 2018; Cassuto et al., 2020; Cassuto et al., 2023), all originating from the same cohort of THA patients and healthy subjects and presented in the context of the major phases of endochondral bone repair (Einhorn and Gerstenfeld, 2015; Marsell and Einhorn, 2011).

Results in short: VEGF-A, VEGFR1, FGF-2 and PDGF-BB were significantly higher, whereas VEGF-C was significantly lower, in presurgery OA patients versus healthy subjects. VEGF-A, VEGFR1 and FGF-2 returned to the level of healthy at 6 W while VEGF-C and PDGF-BB were normalized on day 1 postsurgery. Plasma levels of biomarkers in THA patients increased at 1Y (VEGF-A, VEGF-D), 2 Y (VEGF-C, PDGF-BB) or 5Y (FGF-2) to a level significantly above healthy individuals before returning to control levels between 13Y and 15Y post-surgery, except for VEGF-D that returned to control level at 7Y post-implant. VEGFR1 was significantly above healthy in the immediate aftermath of surgery before returning to the level of healthy and remaining there throughout the study. PIGF did not differ from healthy subjects at any point during the follow-up.

## 4 Discussion

### 4.1 Age-related changes of angiogenic factors

The only angiogenic factor of the current study to change with increasing age was VEGFR2 that significantly decreased with increasing age while VEGF did not show any significant age-related changes (Table 3). Reduced levels of VEGFR2, the main receptor of VEGF-A, may affect the formation of new blood vessels during the process of bone repair and wound healing in ageing individuals which is in line with a study in ageing mice that showed a significant decrease in age-related VEGFR2 expression (Baffert et al., 2004).

### 4.2 Osteoarthritis

Inflammatory changes in the OA joint are accompanied by increased numbers of macrophages in the synovial tissue that secrete proangiogenic factors that are key to the invasion of blood vessels into the avascular cartilage and a prerequisite for the ensuing degenerative changes of OA (Mapp and Walsh, 2012). The current study showed that VEGF-A in presurgery OA patients was significantly higher than in healthy controls which conforms with previous studies showing VEGF-A to increase in knee joints of OA patients (Pfander et al., 2001) and in articular cartilage, subcondral bone, meniscus and synovium of post-traumatic mouse OA (Nagao et al., 2017). The involvement of VEGF-A in OA pathology was confirmed by a study showing that intraarticular injections accelerated cartilage degeneration in rat OA, whereas intraarticular VEGF-A antibody treatment reduced OA progression (Nagao et al., 2017). One of the mechanisms by which VEGF-A is believed to induce OA pathology is through the release of cartilage degradative MMP enzymes (Pufe et al., 2004), which is in line with a previous report in OA patients awaiting THA (Cassuto et al., 2020). Another mechanism by which VEGF-A could induce OA is through inhibition of the synthesis of two main components of articular cartilage, aggrecan and type II collagen (Chen et al., 2012). The current study also showed VEGFR1, but not VEGFR2, to be significantly higher in OA patients awaiting THA versus healthy subjects. The main function of VEGFR1 in OA is thus to serve as a decoy receptor that reduces VEGF-A activity (Hu and Olsen, 2016a) and limits its damaging effects on the articular cartilage as was shown in mice with chemical OA (Matsumoto et al., 2008). Another notable finding of the current study was that VEGF-C in presurgery OA patients was significantly lower than that of healthy controls. The importance of decreased VEGF-C for the pathology of OA was highlighted by a study showing impaired synovial lymphatic system (SLS) function leading to decreased joint clearance in mice with age-related OA due to decreased VEGF-C and VEGFR3 signaling (Lin et al., 2023). Following a 12-week intra-articular administration of VEGF-C, a significant improvement of synovial lymphatic clearance was seen along with increased cartilage area and reduced staining by cartilage-degradative MMP-13 (Lin et al., 2023). The importance of VEGF-C for the maintenance of healthy joints was further confirmed in a chronic model of mice OA showing that intraarticular injections of VEGF-C were able to significantly increase the number of lymphatic vessels thereby improving lymph drainage and containing articular inflammation and bone/cartilage loss (Zhou et al., 2011). PDGF-BB was shown in the present study to be significantly higher in presurgery OA than in healthy subjects, which would suggest that it is part of OA pathology or a counter-measure. Preosteoclasts harvested from destabilized knee joints of mice overexpressing PDGF-BB were shown to develop spontaneous OA-like arthritis characterized by subcondral bone angiogenesis and subsequent joint degeneration which lends support to PDGF-BB being a pathogenic factor in OA (Su et al., 2020). However, the latter study was contradicted by studies in rats showing that intraarticular injections of PDGF-BB reduced cartilage degeneration in OA by inducing increased synthesis of type II collagen, a major component of articular cartilage, and by downregulating biomolecules that induce chondrocyte and matrix loss, such as type X collagen, MMPs and ADAMTS (Cai et al., 2023; Zhu et al., 2021). These opposing effects of PDGF-BB in OA pathology need to be addressed by future studies. The current study also showed that presurgery OA patients had significantly higher levels of FGF-2 than healthy subjects which is in accordance with a previous study showing highly upregulated expression of FGF-2 in synovial tissue and fluid of arthritic joints with higher concentrations correlating with increased disease severity (Ellman et al., 2008). The latter study also reported FGF-2 to be associated with upregulation of matrix degrading enzymes that participate in joint destruction and inhibition of type II collagen and proteoglycan synthesis (Ellman et al., 2008). In contrast, FGF-2 was shown in another study to have a chondroprotective role in human articular cartilage by suppressing the synthesis ADAMTS-4 and ADAMTS-5 (Sawaji et al., 2008) which suggests that additional studies are required to decide its role in OA.

### 4.3 Bone healing of hip implants

The four phases of endochondral bone repair of the current study and their duration are based on the spatiotemporal responses of biomarkers that are characteristic of the various phases of endochondral repair previously described (Einhorn and Gerstenfeld, 2015; Marsell and Einhorn, 2011). As the current study is based on the same population of patients and controls as our previous reports (Cassuto et al., 2018; Cassuto et al., 2020; Cassuto et al., 2023), it is our aim to discuss results, when advantageous for clarity, against the backdrop of our previous observations.

#### 4.3.1 Vascular endothelial growth factors and their receptors

##### 4.3.1.1 VEGF-A

The VEGF family includes six members, VEGF-A, VEGF-B, VEGF-C, VEGF-D, VEGF-E and PIGF. VEGF-A has several isoforms with VEGF_165_ being the most abundant and predominant (Mantsounga et al., 2022) and the isoform analyzed in the current study. VEGF-A acts on two receptors, VEGFR1 (sFlt-1) and VEGFR2 (KDR/sFlk-1). VEGFR2 is the main signaling receptor that is primarily expressed on endothelial cells and upon activation promotes angiogenesis by inducing endothelial cell migration and proliferation, whereas VEGFR1 is primarily a decoy receptor that regulates the activity and functions of VEGFR2 (Mantsounga et al., 2022). The significantly higher level of VEGF-A in presurgery OA patients of the current study versus healthy individuals would have been expected to normalize immediately after the removal of the diseased osteoarthritic hip joint, had it been solely part of OA pathology (Mapp and Walsh, 2012; Pfander et al., 2001; Nagao et al., 2017). However, VEGF-A was shown to increase further on day 1 postsurgery which is in line with previous observations showing high levels of VEGF in the hematoma after bone trauma (Hu and Olsen, 2016a). VEGF-A is released into the hematoma from damaged bone matrix, platelets and immune cells to act as a potent chemoattractant that facilitates the entry of neutrophils into the trauma site where they participate in the microbial defence and removal of damaged tissue (Hu and Olsen, 2016a). A notable observation of the current study was that the augmented level of VEGF-A shortly after the surgical bone trauma was associated with a corresponding increase by its receptor VEGFR1, but not VEGFR2, thus suggesting that the transitory elevation of VEGF-A in the immediate aftermath of bone trauma is primarily aimed at attracting neutrophils to the hematoma rather than being involved in angiogenesis (Hu and Olsen, 2016a). The latter assumption is supported by studies showing VEGFR1 to induce an inflammatory phenotype (M1) by binding to theVEGF-A_165b_ subtype that recruits macrophages toward the site of injury while at the same time inhibiting angiogenesis, whereas VEGFR2 binds to VEGF-A_165a_ that stimulates angiogenesis (Mantsounga et al., 2022). Another important role of VEGF-A in the immediate aftermath of bone trauma is to increase the permeability of the bone marrow and facilitate the migration into the hematoma of mesenchymal stem cells (MSC) that later transform into chondrocytes and osteoblasts (Hu and Olsen, 2016a). This short-lasting phase of elevated VEGF-A after the surgical bone trauma was followed by a sharp decrease to the level of healthy individuals lasting between 6 W and 6 M post-implant. The latter phase of normalized VEGF-A represents the phase when proliferative chondrocytes accumulate and synthesize cartilage in THA patients as shown by high levels of cartilage formation markers (Cassuto et al., 2020) (Fig. 4) thus being in line with a study showing weak VEGF-A expression in proliferative chondrocytes involved in cartilage synthesis (Horner et al., 1999). In accordance, inhibition of VEGF-A was shown to induce chondrogenesis (Hu and Olsen, 2016a) thus confirming the link between low VEGF-A and high cartilage formation. During the following phase of bone repair, the proliferative cartilage transforms into the hypertrophic cartilage that is gradually calcified (Einhorn and Gerstenfeld, 2015; Marsell and Einhorn, 2011). In the current study, a significant increase by VEGF-A began 2 Y post-implant which is in line with a previous study showing high expression of VEGF-A in hypertrophic chondrocytes and high levels of VEGF-A contributing to the death of hypertrophic chondrocytes through the actions of VEGFR2 (Hu and Olsen, 2016a). VEGF-A reached a spatiotemporal peak at 5 Y post-implant which coincided with the peak of VEGFR2 lending strong support to the latter being the primary signaling receptor for VEGF-A during angiogenesis and cartilage remodeling (Hu and Olsen, 2016a). A prerequisite for vascular invasion of the avascular hypertrophic cartilage is that the cartilaginous matrix is degraded by enzymes that create channels through which blood vessels can protrude. VEGF-A is a potent chemotactic factor that promotes the influx of immune cells and osteoclasts into the hypertrophic cartilage and triggers their release of enzymes that degrade the basal membrane and matrix and enable blood vessels to invade and transport osteoblasts into the lacunae left behind by apoptotic chondrocytes (Gerber et al., 1999). This critical connection between VEGF-A and enzymes during cartilage remodeling is supported by the coinciding plasma peaks of VEGF-A/VEGFR2 and multiple enzymes (MMP-8, MMP-9, ADAMTS 4/5, neutrophil elastase, protease 3) at 5 Y post-implant in the present cohort of hip arthroplasty patients (Cassuto et al., 2020) (Fig. 4). The interplay between VEGF-A and cartilage remodeling in the current cohort of hip arthroplasty patients was further supported by the conjoined peaks of VEGF-A and CTX-II, a marker of cartilage degradation biproducts (Cassuto et al., 2020) (Fig. 4). Although VEGF-A is only one of several angiogenic factors involved in the vascularization of the hypertrophic cartilage during endochondral bone repair, it is by far the most important factor, as shown by a total inhibition of angiogenesis following administration of a VEGF-A blocker (Gerber et al., 1999). Moreover, recruitment of chondroclasts that release MMP-9 was decreased following blockage of VEGF-A (Gerber et al., 1999). MMP-9 has been subject to extensive studies and shown to be present at high concentrations in preosteoclasts and osteoclasts at the cartilage-bone interface during cartilage remodeling thus suggesting that the enzyme regulates the bioavailability of VEGF-A which would explain the delayed vascular invasion in MMP-9-deficient animals (Colnot et al., 2003). The latter assumption was supported by showing that administration of recombinant VEGF-A to MMP-9-deficient animals was able to restore the recruitment and activation of chondroclasts, osteoblasts and endothelial cells that vascularize and remodel the cartilage, as shown by reduced amount of hypertrophic cartilage parallel to increased synthesis of bone matrix (Colnot et al., 2003). However, in mice lacking MMP-9, other MMPs may contribute to endochondral ossification (Ortega et al., 2010) which supports a compensatory interaction between enzymes released from osteoclasts during cartilage remodeling and could shed light on the multitude of enzymes shown to reach high plasma levels during cartilage remodeling in patients receiving hip implants (Cassuto et al., 2020) (Fig. 4). Another noteworthy observation of the latter study (Cassuto et al., 2020) was that MT1-MMP (MMP-14), a cell surface enzyme, was shown to be the sole MMP to increase in the immediate aftermath of the surgical bone trauma and to remain significantly above the level of healthy subjects throughout the formation and remodeling of the cartilage callus. The latter observation is in line with previous studies showing MT1-MMP, along with MMP-9, to play a central role in several steps of angiogenesis, such as sequestration of VEGF-A from the ECM, regulation of VEGFR2, endothelial cell migration, formation of capillary tubes, and crosstalk with other angiogenic factors (Eisenach et al., 2010; Genis et al., 2006). In confirmation, mice deficient of MT1-MMP were shown to have reduced numbers of proliferative chondrocytes, absence of vascular invasion of the hypertrophic cartilage and inadequate remodeling of the ECM (Genis et al., 2006). However, not all enzymes released during cartilage remodeling are pro-angiogenic as some, like neutrophil elastase (NE) and ADAMTS5, reaching high levels during the cartilage remodeling phase of hip arthroplasties (Cassuto et al., 2020) (Fig. 4), act as anti-angiogenic factors by inducing degradation or modification of VEGF-A (Ai et al., 2007; Sun et al., 2015) which reduces its ability to bind to the VEGFR2 receptor (Kurtagic et al., 2009).

Other mediators shown to reach their peaks in conjunction with VEGF-A during endochondral cartilage remodeling in THA patients are the proinflammatory cytokines, IL-8 and IL-1β (Cassuto et al., 2018) (Fig. 4). IL-8 has been recognized as a potent pro-angiogenic factor that acts by stimulating VEGF-A production and VEGFR2 activation in endothelial cells (Martin et al., 2009). IL-8 has also been shown to directly enhance endothelial cell survival, proliferation and capillary tube organization while at the same time inhibiting endothelial cell apoptosis (Li et al., 2003). Furthermore, IL-8 is a potent chemoattractant for neutrophils and macrophages that accumulate at the site of bone repair and release their contents of enzymes (Cassuto et al., 2020; Li et al., 2003) which are critical for neoangiogenesis to proceed normally (Colnot et al., 2003; Eisenach et al., 2010; Genis et al., 2006). Such a connection is supported by patient data from the current cohort of THA patients showing plasma peaks of IL-8 and IL-1β to coincide with high plasma levels of multiple enzymes involved in cartilage remodeling (Fig. 4). In similarity with IL-8, IL-1β been shown to have wide-ranging effects on angiogenesis by driving the transcription of both VEGF-A and VEGFR2 in myocytes and endothelial cells (Fahey and Doyle, 2019). This was demonstrated in IL-1β KO mice showing reduced VEGF-induced recruitment of endothelial progenitor cells leading to reduced neovascularization (Fahey and Doyle, 2019). Another important role of IL-1β in angiogenesis is its ability to induce vascular permeability by regulating cell-to-cell adhesion molecules and tight junctions thereby adding to the permeability enhancing properties of VEGF-A (Bates and Harper, 2002). There is now significant evidence to show that increased vascular permeability is a critical part of angiogenesis and accompanies sprouting during injury repair (Bates and Harper, 2002). The VEGF-A-induced increase in permeability is mediated through stimulation of VEGFR2, not VEGFR1, as shown by the use of specific receptor inhibitors (Bates and Harper, 2002) which conforms with the current study showing VEGFR2 to increase alongside VEGF-A during remodeling of the hypertrophic cartilage, whereas VEGFR1 did not deviate from healthy subjects (Fig. 4). BMP-9 is another mediator that showed a striking spatiotemporal similarity with VEGF-A during bone healing of hip implants (Cassuto et al., 2023). BMP-9 promotes the formation of the cartilagenous callus by stimulating chondrocytes to synthesize aggrecan and type II collagen and by promoting its transformation into the hypertrophic cartilage (Majumdar et al., 2001; van Caam et al., 2015), while at the same time preventing its degradation by acting as a potent anti-angiogenic factor that inhibits VEGF-stimulated angiogenesis (Scharpfenecker et al., 2007). However, BMP-9 and TGF-β1 have also been shown to act as vascular maturation factors that promote cell cycle arrest, formation of cell-to-cell adhesions, recruitment of pericytes and matrix formation (Medina-Jover et al., 2022).

Of note is that the significantly augmented level of VEGF-A persisted until 13 Y post-implant, i.e. beyond the phase of cartilage remodeling and well into the phase of bone formation as shown by high levels of bone formation markers (Cassuto et al., 2018) (Fig. 4). In contrast to persistent augmentation of VEGF-A, VEGFR2 decreased sharply at 7 Y post-implant which aligns with the sharp decrease of the cartilage degradation marker, CTX-II (Cassuto et al., 2020) (Fig. 4). The latter connection is supported by a study showing VEGFR2 only to be detected in osteoclasts and hypertrophic chondrocytes but not in osteoblasts, whereas VEGF-A has also been detected in osteoblasts which supports a role for the latter during the osteogenic phase (Clarkin and Gerstenfeld, 2013). In a study by Hu and Olsen (2016b), the authors showed that conditional deletion of VEGFR2 in osteoblastic lineage cells induced increased maturation of osteoblasts and formation of bone which is in line with the current and previous study in hip arthroplasty patients showing the decreased level of VEGFR2 to coincide with a significant increase by P1NP (N-terminal propeptide of collagen type 1) (Cassuto et al., 2018) (Fig. 4), a marker of type I collagen synthesis that constitutes 95 % of collagen substance in bone tissue and is believed to influence the mineralization and ultimately the strength of bone (Christenson, 1997). Moreover, inhibition or deletion of VEGFR2 in osteoblastic cells increased bone mineralization as shown by increased level of the bone formation marker alkaline phosphatate (ALP) (Hu and Olsen, 2016b), which aligns with the current and previous study in arthroplasty patients showing a normalized level of VEGFR2 to coincide with increased levels of bone specific ALP at 10 Y post-implant (Cassuto et al., 2018) (Fig. 4). The current study also showed the sustained elevation of VEGF-A to coincide with the peak of RANKL at 13 Y post-implant (Fig. 4) which is in line with a study showing VEGF-A and RANKL to stimulate the recruitment, differentiation and activation of osteoclasts during coupled bone remodeling through a common signaling pathway in osteoclasts (Henriksen et al., 2003).

##### 4.3.1.2 VEGF-C and VEGF-D

The role of the lymphatic system during bone repair has only recently begun to emerge by studies refuting the long-held view that bone is devoid of lymphatics (Biswas et al., 2023). Although the vascular and lymphatic systems develop independently, they are structurally and functionally interconnected in ways that are critical for bone repair and homeostasis (Huang et al., 2024). The two lymphangiogenic mediators, VEGF-C and VEGF-D, are secreted as precursors that need proteolytic processing to gain their mature form whereupon they bind and activate both VEGFR2 (KDR/Flk1) and VEGFR3 (Flt4) on lymphatic endothelial cells (Huang et al., 2024) although there is much evidence to support VEGFR3 as being the main mediator of lymphatic growth and maintenance (Veikkola et al., 2001). The current study showed VEGF-C and VEGF-D to increase in concert with their receptors, VEGFR2 and VEGFR3, during the secondary pro-inflammatory phase of bone repair in hip implant patients (Cassuto et al., 2018) (Fig. 4). Lymphatic endothelial cells are normally quiescent in the resting state but respond to inflammatory stimuli by undergoing lymphangiogenesis (Ristimaki et al., 1998) that facilitates interstitial clearance and promotes resolution of inflammation (Huggenberger et al., 2011). A functional relationship between lymphatic drainage and fracture repair was investigated in patients with tibial fractures and showed dilated lymph vessels and enlarged inguinal lymph nodes in patients with fully healed fractures whereas non-union fractures revealed decreased lymph node area (Szczesny et al., 2007) thus supporting the importance of a well-functioning lymphatic vasculature for successful bone repair. In a recent study investigating the effect of lymphatic dysfunction on fracture healing, the authors showed that mice with tibial fractures treated with a VEGFR3 blocker had significantly increased tissue swelling parallel to reduced lymphatic clearance and delayed fracture healing as opposed to vehicle treated controls (Zheng et al., 2024). The latter findings lend support to the importance of a concomitant augmentation of the lymphangiogenic mediators, VEGF-C and VEGF-D, and their main target receptor, VEGFR3, during fracture healing of hip implants (Fig. 4). However, in addition to restored lymph flow, VEGF-C and VEGF-D are also known for their osseoinductive role in bone formation and homeostasis. A notable observation of the current study was that VEGF-D dropped sharply to the level of healthy individuals at 7 Y post-implant, signaling a role confined to the early phase of osteoblast differentiation during spongious bone formation which is in line with a previous study showing VEGF-D to promote osteogenic differentiation of osteoblasts via activation of VEGFR3 (Orlandini et al., 2006). In contrast, VEGF-C remained significantly above healthy individuals until 13 Y post-implant thus coinciding with markers of both osteoblast and osteoclast formation in hip implant patients (Cassuto et al., 2018; Cassuto et al., 2020; Cassuto et al., 2023) (Fig. 4) which is in line with a study showing activation of VEGFR2 and VEGFR3 to induce osteogenic differentiation of human MSCs (Murakami et al., 2017). The augmented level of VEGF-C in arthroplasty patients at 13 Y post-implant was also shown to coincide with peak plasma level of RANKL (Cassuto et al., 2018) which is in alignment with a study showing VEGF-C to stimulate RANKL-mediated osteoclastic bone resorption through a VEGFR3-mediated pathway and a reciprocal RANKL-induced release of VEGF-C through an autocrine mechanism (Zhang et al., 2008).

##### 4.3.1.3 Placental growth factor (PIGF)

PIGF was the second member of the VEGF family to be identified in the placenta and shown to bind exclusively to VEGFR1 (De Falco, 2012). Despite high expression in the placenta, a PIGF KO model in mice showed that absence of PIGF did not have deleterious effects on physiological development in mice, making it dispensable (De Falco, 2012). However, deletion of PIGF in the adult mice during pathological conditions significantly impaired angiogenesis suggesting that the activity of PIGF is confined to pathological processes (De Falco, 2012). The latter is in line with the current study by showing that PIGF remained at the level of healthy subjects throughout the two-decade follow-up of physiologic bone repair in hip arthroplasty patients.

#### 4.3.2 PDGF-BB

PDGF has several isoforms that are produced by platelets, skeletal and smooth muscle cells, leucocytes, macrophages, and vascular endothelial cells. The isoform PDGF-BB is secreted as active ligand that attaches to two receptor subtypes, PDGFR-α and PDGFR-β, located on the surface of various cell types, such as vascular endothelial cells, osteoclasts, osteoblasts and fibroblasts (Graham et al., 2009). The α- receptor is mainly expressed by chondrocytes during cartilage callus remodeling whereas the β-receptor is expressed throughout fracture healing (Graham et al., 2009). PDGFRs are expressed on cell surfaces during inflammatory processes (Graham et al., 2009) which aligns with our findings in hip arthroplasty patients showing augmented levels of PDGF-BB during the primary and secondary phase of inflammation (Cassuto et al., 2018) (Fig. 4). PDGF-BB is considered the most prevalent and important among the different isoforms due to its physiological actions and ability to bind to all known receptor isotypes, although it binds with significantly greater affinity to PDGFR- β (Graham et al., 2009). PDGF-BB has been shown to be of critical importance for VEGF- and FGF-induced neovascularization of the hypertrophic cartilage during bone repair which is characterized by endothelial cells breaching the basement membrane to form EC-lined capillary tube networks known as “sprouts” (Caplan and Correa, 2011). EC-derived PDGF-BB is considered to be the primary driver of the migration and proliferation of mural cells known as “pericytes” that enwrap the abluminal surface of the EC tubes and stabilizes them until they mature into lumenized blood vessels (Caplan and Correa, 2011). The importance of PDGF-BB was confirmed by studies in mice showing that disruption of the ligand or its receptor, PDGFR-β, will reduce, not block, the accumulation of pericytes within the capillary beds (Kemp et al., 2020), thus suggesting that multiple factors are involved in pericyte recruitment. One such factor is TGF-β1 that shows a striking spatiotemporal similarity to PDGF-BB in the current population of hip arthroplasty patients (Cassuto et al., 2023). The importance of the a coordinated increase by the two mediators was confirmed in a study showing that TGF-β1 pretreatment of pericytes induced a substantially greater pericyte invasion in response to PDGF-BB and a subsequent acceleration of capillary assembly (Kemp et al., 2020).

#### 4.3.3 FGF-2

The pro-angiogenic role of FGF-2 or basal FGF on endothelial cells is multifaceted and include modulation of endothelial cell proliferation, migration, release of proteases and intercellular gap junction communication (Presta et al., 2005). FGF-2 interacts with FGF receptor 1 (FGFR1), which is the main FGF receptor on endothelial cells, but can also interact with FGFR2 expressed in small amounts on endothelial cells (Presta et al., 2005). Activation of FGFR1 triggers migration, proliferation, protease production and tubular morphogenesis whereas FGFR2 activation is limited to cell mobility (Presta et al., 2005). Stimulation of endothelial cells by FGF-2 will induce chemotaxis of ECs and the release of MMPs and serine proteases at the forefront of EC migration that will promote disruption of the basal membrane and ECM during angiogenesis (Presta et al., 2005). A significant crosstalk exists between FGF-2 and members of the VEGF family and their receptors during angiogenesis and lymphangiogenesis as shown by inhibition of both FGF-2 and VEGF-induced angiogenesis following administration of VEGFR2 antagonists and by up regulation of VEGFRs and FGFRs in endothelial cells by FGF-2 (Presta et al., 2005). The lymphangiogenic effects of FGF-2 are mediated by VEGF-C and VEGF-D through activation of VEGFR3 (Presta et al., 2005). However, FGF-2-KO mice are morphologically normal and show no differences in neovascularization following injury compared to normal animals which supports a redundant regulation of angiogenesis (Presta et al., 2005). The current study showed FGF-2 to increase sharply at 5Y post-implant and return to the level of healthy volunteers at 13Y post-surgery. The peak of FGF-2 at 5 Y post-THA coincided with peak levels of MMPs, ADAMTS, and serine proteases along with high levels of cartilage degradation products (Cassuto et al., 2020) (Fig. 4) which is in line with previous studies showing FGF-2 to stimulate the release of serine proteases (Presta et al., 2005) and MMPs (Ellman et al., 2008; Ardi et al., 2009). However, not all enzymes shown to be significantly augmented during cartilage remodeling in THA patients (Fig. 4) are pro-angiogenic. The pro-angiogenic role of TIMP1-free-MMP-9 (Cassuto et al., 2020) is omnipotent and shown to induce a significant increase in the bioavailability of FGF-2 thereby leading to increased angiogenesis (Ardi et al., 2009) whereas significantly increased levels of neutrophil elastase (Cassuto et al., 2020) (Fig. 4), has been shown to degrade VEGF-A and FGF-2 and curtail their pro-angiogenic effects (Ai et al., 2007) thereby being part of the mechanisms that regulate angiogenesis.

## 5 Clinical and practical applications

The inherent ability of damaged bone, either accidentally or surgically, to regenerate is a critical part of bone biology and our ability to continuously maintain the skeleton. Angiogenesis that induces and regulates the capillary invasion into the hypertrophic cartilage is one of the main pillars of endochondral bone repair without which the whole process will grind to a halt (Hu and Olsen, 2016a). In the current study we showed for the first time in humans that vascular and lymphatic angiogenesis occur simultaneously through activation of VEGFR2 and VEGFR3 and that these actions are associated with augmented levels of FGF-2 and PDGF- BB that together dilate existing blood vessels in the bone-cartilage interface and enzymatically degrade their basal membrane and adjacent cartilage which enables the migration of endothelial cells and the formation of mature capillaries (Hu and Olsen, 2016a). A notable observation was that biomarkers in THA patients shown to regulate the complex and coordinated actions of proinflammatory cytokines, cartilage degradative enzymes, pro-angiogenic molecules, and osteogenic factors during various phases of osseointegration (Cassuto et al., 2018; Cassuto et al., 2020; Cassuto et al., 2023) (Fig. 4) showed remarkable spatiotemporal similarities with biomarkers investigated in experimental studies of endochondral bone repair (Einhorn and Gerstenfeld, 2015; Marsell and Einhorn, 2011), albeit with a significant elongation of the healing process. These similarities suggest that a preformed template for endochondral bone repair is activated following the surgically induced bone trauma in THA patients but requires significantly longer time to reach complete osseointegration due to superimposed external factors that are likely to interfere with the normal process of bone repair. One such factor is excessive micromotion between the implant and surrounding bone tissue due to weight bearing and mechanical properties of shaft materials (Wazen et al., 2013). Another important factor is the release of implant-derived toxic wear debris into the site of bone repair that is likely to consume much of important immune cell function that is required for bone healing (Goodman et al., 2019) while at the same time negatively affecting the function of bone forming cells (Zhang et al., 2020). Host-related factors such as age, nutrition, medications, diseases and smoking that impede the bone could also contribute to a slow progress, of otherwise normal, endochondral repair (Grzeskowiak et al., 2020).

Bone repair following THA has been widely assumed to reach the final stages of osseointegration within 2 years of the index operation based primarily on perceived early stiffness of periimplant tissues and radiographic evidence of calcification around the implant shaft. However, in view of the critical role of angiogenesis in the progression of endochondral repair and the current study showing pro-angiogenic mediators to peak at 5 Y post-implant makes the above timeline of complete osseointegration of hip implants within 2 years rather unlikely. In addition to increased knowledge and understanding of the biomolecular mechanisms that regulate normal fracture repair, the current study sheds important and critical light on the biomolecular mechanisms that govern successful osseointegration of hip implants. This is particularly important for the understanding and identification of the role of specific biomolecules in the processes that could cause the implant to fail during its lengthy healing. As our lab possesses blood samples from several other longitudinal THA studies, we have been able to gradually analyze biomarkers from accumulating numbers of failed implants that will enable us to elaborate on differences in the biomolecular patterns between successfully integrated implants and implants that fail. This knowledge, to be presented, will contribute to the identification of important mechanisms of implant failure and hopefully aid in the research and development of new drugs that target specific biomarkers (proteins) that have been identified as disease-driving factors (Shepard et al., 2017).

### 5.1 Limitations

There are limitations to the current study. One is that only limited demographic and drug use data are reported in the THA and healthy cohorts. Moreover, we did not report or exclude patients on drugs other than those known to affect bone metabolism, such as steroids, immunotherapy, or bone-regulating drugs (e.g., bisphosphonates, denosumab, calcitonin, PTH). Three patients on prohibited drugs (bisphosphonates) were identified, albeit excluded on other criteria. Aside from a history of recent bone trauma, no explicit medical and drug use data, other than that being used for exclusion of THA patients, were considered when recruiting healthy subjects. Second, we measured biomarkers in plasma that do not only reflect changes related to the implant as they may also originate from other parts of the musculoskeletal system. We believe however that the latter changes would be equally reflected in the population of gender/age-matched healthy controls. Third, recruitment of healthy controls and OA patients awaiting THA was done between 10Y and 13Y after ending inclusion of THA patients into the study. A particular strength of the study is that biomarkers were sampled for two decades in a single population of arthroplasty patients and analyzed on a high-sensitivity and wide dynamic range platform allowing for detection of minor changes with high accuracy.

## CRediT authorship contribution statement

**Jean Cassuto:** Writing – original draft, Resources, Software, Investigation, Formal analysis, Visualization, Supervision, Methodology, Funding acquisition, Conceptualization, Validation, Project administration, Data curation. **Agnetha Folestad:** Funding acquisition, Conceptualization, Writing – review & editing. **Jan Göthlin:** Formal analysis, Writing – review & editing, Methodology. **Henrik Malchau:** Writing – review & editing, Investigation, Conceptualization, Resources, Funding acquisition, Methodology, Data curation. **Johan Kärrholm:** Writing – review & editing, Supervision, Methodology, Formal analysis, Validation, Project administration, Funding acquisition, Data curation, Resources, Visualization, Software, Investigation, Conceptualization.

## Declaration of competing interest

The authors declare the following financial interests/personal relationships which may be considered as potential competing interests: Jean Cassuto reports a relationship with Sahlgrenska University Hospital Mölndal Hospital that includes: employment and non-financial support. If there are other authors, they declare that they have no known competing financial interests or personal relationships that could have appeared to influence the work reported in this paper.

## Data Availability

Data will be made available on request.
